# Prospects for anti-*Candida* therapy through targeting the cell wall: A mini-review

**DOI:** 10.1016/j.tcsw.2021.100063

**Published:** 2021-10-16

**Authors:** Sanaz Ahmadipour, Robert A. Field, Gavin J. Miller

**Affiliations:** a Department of Chemistry and Manchester Institute of Biotechnology, The University of Manchester, Manchester M1 7DN, United Kingdom; b Iceni Diagnostics Ltd, The Innovation Centre, Norwich Research Park, Norwich, Norfolk NR4 7GJ, United Kingdom; c Lennard-Jones Laboratory, School of Chemical and Physical Sciences, Keele University, Keele, Staffordshire ST5 5BG, United Kingdom

**Keywords:** Fungal cell wall, Chitin, Glycoproteins, Inhibitors, Vaccines

## Abstract

The impact of fungal infections on humans is a serious public health issue that has received much less attention than bacterial infection and treatment, despite ever-increasing incidence exacerbated by an increased incidence of immunocompromised individuals in the population. *Candida* species, in particular, cause some of the most prevalent hospital-related fungal infections. Fungal infections are also detrimental to the well-being of grazing livestock, with milk production in dairy cows, and body and coat condition adversely affected by fungal infections. Fungal cell walls are essential for viability, morphogenesis and pathogenesis: numerous anti-fungal drugs rely on targeting either the cell wall or cell membrane, but the pipeline of available bioactives is limited. There is a clear and unmet need to identify novel targets and develop new classes of anti-fungal agents. This mini review focuses on fungal cell wall structure, composition and biosynthesis in *Candida* spp., including *C. auris*. In addition, an overview of current advances in the development of cell wall targeted therapies is considered.

## Introduction

The widespread use of antifungals in agricultural and clinical settings has led to the emergence of drug-resistant fungal infections, which represent major threats to food security and human health (Nguyen et al., 2021). Despite the high mortality and morbidity rate of invasive fungal infections (IFIs), treatment options are limited and the anti-fungal drug discovery pipeline is under developed (Perfect, 2017). There are three main classes of available anti-fungal agents to treat IFI: the first class of antifungal agents, azoles (e.g. fluconazole, itraconazole) inhibit the ergosterol biosynthesis of fungal cell wall. There is a significant overlap in the use of azoles to treat both human and plant fungal pathogens. They are widely used in grain- and grass-growing environments, as well as clinical settings, to control *Candida* spp., *Aspergillus* spp. and *Cryptococcus* spp. infections (Azevedo et al., 2015). The second class of antifungal agents, polyene amphotericin B-deoxycholate bind directly to ergosterol embedded in the fungal cell membrane, leading to cell lysis. The third class, echinocandins (caspofungin, micafungin, anidulafungin and rezafungin), inhibit fungal β-(1,3)-D-glucan cell wall biosynthesis (Gow et al., 2017). Along with those three classes of antifungal agents, the pro-drugs and 5-fluorocytosine/ flucytosine (FLC), an antimetabolite, act as antifungal agents. FLC, within susceptible fungal cells, is converted into fluorouracil by cytosine deaminase (CD), resulting in inhibition of fungal RNA and DNA synthesis (Nechipadappu et al., 2018).

Discovering new drugs for the treatment of resistant IFIs is challenging due to difficulties with poor drug uptake, limiting access to intracellular targets (Perlin, 2007). Herein we review the cell wall composition across *Candida* species and opportunities associated with fungal cell wall and associated targets.

## Fungal cell wall overview for *Candida* spp.

**Fig. 1:**
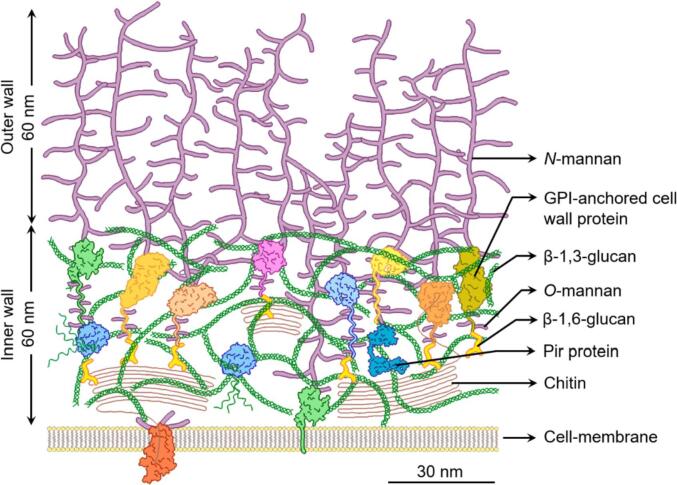
Structural organization of the *Candida albicans* cell wall [taken from (Lenardon et al., 2020)] using open access article under creative commons attribution (CC-BY 4.0) license].

The complex fungal cell wall is vital for maintaining cellular shape and integrity (Garcia-Rubio et al., 2020). Fungi have a cell wall comprising of two layers (inner and outer), which are visible by electron microscopy. The composition of the cell wall is subject to change and may vary within a single fungal isolate, depending upon the conditions and stage of growth, and where glycoprotein, glucan and chitin components are extensively cross-linked together to form a complex network (Fig. 1) (Lenardon et al., 2020), forming the overall structural basis of the cell wall (Gow and Hube, 2012).

### Inner cell wall compositions for *Candida* spp

**Fig. 2:**
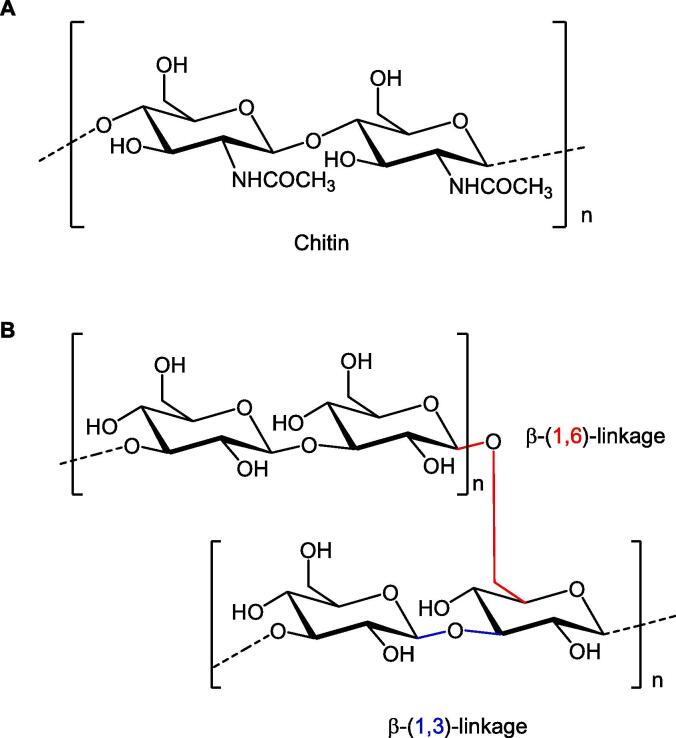
Chemical structure of polysaccharides associated with fungal cell wall. A) Chitin B) β-(1,6)-branched β-(1,3)-glucan.

In most fungal species, the inner cell wall is composed of chitin and β-glucans (β-1,3 and β-1,6 linked polymers of glucose) that are linked to chitin *via* β-(1,4) linkages (Lenardon et al., 2020). Chitin is a linear chain of β-(1,4)-*N*-acetylglucosamine and represents the dominant structural polysaccharide in the fungal cell wall (Fig. 2). The major synthases that make chitin (and glucans) reside in the plasma membrane (PM) and use UDP-sugars (Ahmadipour and Miller, 2017) as the sugar donors for the formation of a polysaccharide, which is extruded into the cell wall (Bowman and Free, 2006, Gow et al., 2017). The sugar nucleotide uridine diphosphate (UDP)-*N*-acetylglucosamine (from the cytoplasmic side of the membrane) is the substrate for chitin synthase which is responsible for the synthesis of chitin with molecular weights from 100 to 130 KDa observed for the final polysaccharide. The gene encoding β-(1,3)-D-glucan synthase, *FKS1* and *FKS2*, were initially identified in *Saccharomyces cerevisiae* (Bowen et al., 1992).

### Outer cell wall composition for *Candida* spp.

The outer cell wall in *Candida* spp., including *C. albicans*, consists of layers of highly glycosylated cell wall proteins that are decorated with *N*- and *O-*linked terminal mannans and phosphomannans (Fig. 1). *N*-Linked glycans often display extensive branching, while *O*-linked glycans tend to be linear chains of two to six mannose residues and act as a mask to hide other glycans (i.e. β-1,3 glucan) (Garcia-Rubio et al., 2020). Outer mannan chains do not influence the overall cell shape as they are less rigid compared to β-glucans and chitin. However, they impart low permeability and porosity to the cell wall, which reduces antifungal drug uptake by affecting the resistance of the cell wall to attack by host molecules and the permeability of the wall to antifungal drugs, without influencing cell shape (Gow and Hube, 2012). Pathogen recognition by the innate immune system is crucial to initiate efficient protective immune responses during the fungal infection. Pathogen associated molecular patterns (PAMPs) are located on the cell wall. Most fungi have *O-* and *N*-Linked mannosylated PAMPs, considered as PAMP ligands that are recognized by a range of C-type lectin receptors (CTLs) (Vendele et al., 2020). C-type lectin receptors are a family of pattern recognition receptors (PRR) that recognize the structure of the cell surface polysaccharides. Multiple CTLs are involved in pathogen recognition of fungal cell wall components including β-glucan, chitin, mannan and melanin. Dectin-1 recognizes β-1,3-glucan, the conserved element of the inner cell wall of most of fungal pathogens. Manno-proteins mask β-glucan layer decreasing the recognition of fungi by the host immune system, a process that is mediated by Dectin-1, impacting the capacity of the host phagocytic cells to uptake and kill *Candida* cells (Brown et al., 2002). However, fungal mannans are more complex than glucan, comprising linear and branched polymer of mannose linked *via* α-1,2, α-1,3, α-1,4, α-1,6 and β-1,2 glycosidic bonds that may further modified by phosphodiester side chain.

### Cell wall composition across *Candida* spp

Analysis of cell wall composition across a range of *Candida* species shows largely similar levels of chitin, mannan and glucan, but some significant differences are also apparent: *C. krusei* and *C*. *auris* show a higher content of chitin when compared to other species, but are also different compared to one another (Table 1). Mannan content was noticeably higher in *C. guilliermondii*, with lower levels of glucan. In *C*. *albicans, C. krusei, C. tropicalis, and C. auris*, *O*-linked mannans comprised about 15% of the total content of cell wall mannan but in *C*. guilliermondii this percentage was over twice that amount. All species showed similar levels of cell wall protein, except *C*. *krusei* which showed lower levels. Cell wall porosity was significantly lower in the walls of *C. albicans* and *C. auris* (Navarro-Arias et al., 2019).

**Table 1:** Analysis of cell wall composition in *Candida* species (Navarro-Arias et al., 2019).

Cell wall abundance						
Organism	Chitin (%)	Mannan (%)	Glucan (%)	Protein (µg)^a^	Porosity^c^ (%)	Phosphomannan content (µg)^b^
*C. albicans*	2.0 ± 1.3	36.1 ± 4.2	61.7 ± 4.4	146.4 ± 14.9	28.4 ± 8.7^d^	114.2 ± 14.3
*C. krusei*	8.2 ± 1.0^d^	23.9 ± 2.0^d^	67.8 ± 2.0	76.4 ± 14.8^d^	57.7 ± 10.5	66.1 ± 9.0
*C. auris*	4.5 ± 1.6^d^	30.8 ± 1.1	64.9 ± 2.5	138.7 ± 18.2	30.9 ± 9.4^d^	110.1 ± 15.2
*C. tropicalis*	2.3 ± 1.0	36.2 ± 3.5	62.9 ± 5.0	129.7 ± 15.3	62.8 ± 8.9	139.5 ± 19.0^d^
*C.guilliemondii*	2.2 ± 1.0	47.8 ± 4.0^d^	50.0 ± 5.0^d^	131.6 ± 18.9	72.3 ± 12.7	131.2 ± 8.9^d^

Notes: ^a^µg of protein/mg of cell wall. ^b^µg of alcian blue bound/OD_600_ = 1. ^c^Relative to diethylaminoethyl (DEAE)-dextran. ^d^*P* < 0.05, when compared with the values from the other species analyzed. Data presented as mean ± SD. Cell cultures were propagated at 30 °C in Sabouraud broth (1% [w/v] mycological peptone, 4% [w/v] glucose).

### Biofilm formation in *Candida* spp.

In *Candida* species, biofilm formation and biofilm-associated drug resistance are common, with biofilm formation suggested as a pivotal factor for the persistence of *Candida* in patients and the environment. *Candida* biofilms often resist the effects of available anti-fungal therapies and the complex of mannan and glucan is a key component of the biofilm extracellular matrix (Mitchell et al., 2015). Scanning electron microscopy has shown that the matrix of *C. auris,* an emerging multidrug-resistant pathogenic fungus that can be transmitted from patient to patient as a skin colonizer, is rich in mannan-glucan polysaccharides; the hydrolysis of which reduces drug tolerance. The analyses of the isolated extracellular polysaccharide matrix were carried out by gas chromatography. The amount of polymer observed was generally consistent, but the content was variable (mean ± standard deviation ratio of mannan to glucan = 1.0 ± 0.47). To disrupt the matrix components, the biofilm was pretreated with either an α-mannosidase or a β-glucanase. The results of susceptibility testing on the biofilms with the antifungal (fluconazole) showed that hydrolytic disruption of each matrix polysaccharide increased fluconazole susceptibility for all *C. auris* biofilm isolates (Dominguez et al., 2019). Treatment with α-mannosidase resulted in a 60% decrease in biofilm burden after exposure to fluconazole. Hydrolysis of matrix glucan had less impact, with an average biofilm burden reduction of only 25%. These observations support the role of matrix mannan and glucan in antifungal drug sequestration (Dominguez et al., 2019).

## Biosynthesis of cell wall polysaccharides

### Core polysaccharides: Chitin and β-(1,3)-glucan

**Fig. 3:**
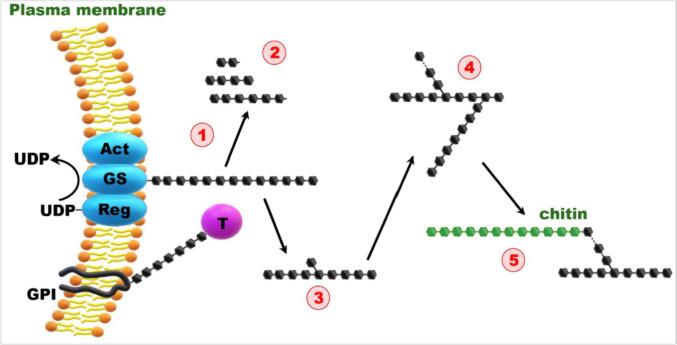
Putative sequential events in the synthesis and remodeling of β-(1,3) glucan.1) Synthesis of linear glucan chains (glucan synthase complex composed of a catalytic [GS], *FKS/GSC*, activating [Act], and *RHO1*, regulating [Reg] subunits). 2) Hydrolysis of glucans. 3) Branching of β-(1,3) glucan. 4) Elongation of β-(1,3) glucan side chains. 5) Cross-linking with branched [β-(1,3)] glucan. Glycosylphosphatidylinositol (GPI)-anchored transglycosidase or hydrolases (T) bound to the membrane can act on the polysaccharides in the cell wall space [re-produced from (Gow et al., 2017) with permission, license number (1131560–1)].

β-(1,3)-Glucans are synthesized at the plasma membrane-bound glucan synthase complex, which uses UDP-glucose on its intracellular side as a substrate and extrudes linear β-(1,3)-glucan chains through the membrane into the cell wall space (Douglas, 2001). After synthesis of linear β-(1,3)-glucan, the polysaccharides can be remodeled through the combined actions of a specific hydrolase and glucosyltransferase to form the modified glucan chains (Fig. 3). This glucan chain becomes cross-linked to different polymers such as chitin, leading to the complex 3D network of polysaccharides in the fungal cell wall (Yoshimi et al., 2016).

In contrast, mannans and other decorating polysaccharides such as α-(1,3)-glucan and β-(1,6)-glucan are synthesized in the endoplasmic reticulum (ER) and Golgi, where they may be conjugated to cell wall proteins and transferred to the cell wall by the secretory pathway (Gow et al., 2017).

### Cell wall proteins

#### Glycosylphosphatidylinositol (GPI) anchors

**Fig. 4:**
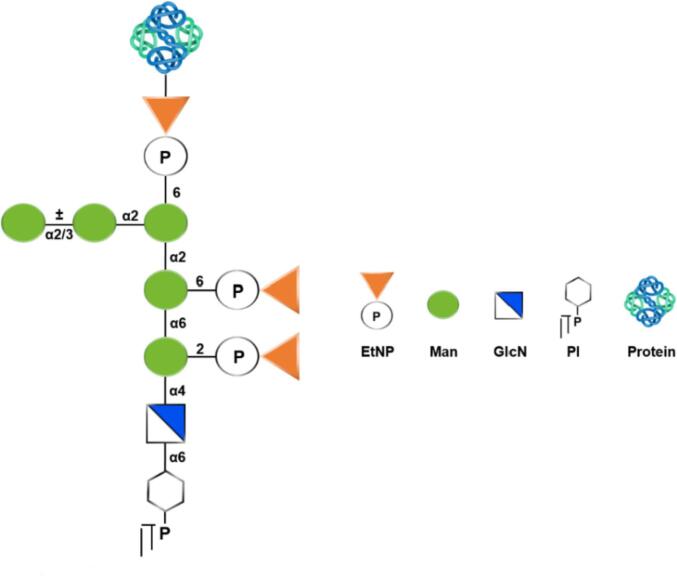
Representative core structure of the Glycosylphosphatidylinositol (GPI) structure from GPI-anchored proteins in *S. cerevisiae*.

Fungal cell walls have multiple proteins with diverse and species-specific functions. They play a role in modifying the cell wall properties, adherence to surfaces and protecting the fungus from harmful environmental elements. GPI-anchors are present in eukaryotes and anchor a wide variety of proteins to the extracellular part of the plasma membrane. In fungi, many of the cell wall synthases, remodeling or degrading enzymes, are attached to the outer plasma membrane *via* a GPI unit (Fig. 4) (Yadav and Khan, 2018). In several fungi, *C. albicans* and *S. cerevisiae* in particular, some of the GPI-anchored proteins (GpiPs) are released from the GPI anchor and covalently linked to β-(1,6)-glucan, thus being transiently localized at the plasma membrane (Richard and Plaine, 2007).

#### Fungal cell wall glycoproteins

Most cell wall proteins are glycoproteins that have passed through the secretory pathway in transit to the cell wall. In *S. cerevisiae* and *C. albicans*, proteins contain roughly 30–50% of the cell wall by dry weight (Bowman and Free, 2006). Glycoproteins are extensively modified with *N*- and *O*-linked oligosaccharides and among fungi, the structure of the oligosaccharide chains attached to these glycoproteins are different. Mannoproteins of the *S. cerevisiae* and *C. albicans* cell walls are glycosylated with chains rich in mannans. In contrast, the glycoproteins of *A. fumigatus* and *N. crassa* have galactomannan, consisting of galactose and mannose residues. Furthermore, some cell wall proteins possess a GPI anchor which is added to proteins containing a C-terminal signal sequence and act to direct and localize these proteins to the plasma membrane and cell wall. Many cell wall proteins are integrated into the wall *via* covalent linkages between the *N*- and *O*- linked site of the sugar and/or in the GPI anchor with those in chitin or glucan (Bowman and Free, 2006).

## Antifungal treatment strategies

### GPI anchor biosynthesis inhibitors

**Fig. 5:**
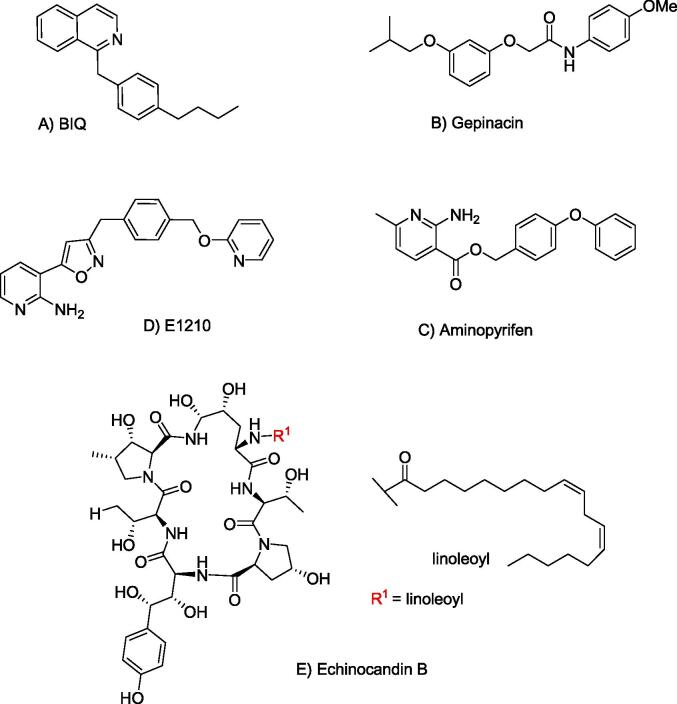
Chemical structure of GPI anchor biosynthesis inhibitors A) BIQ B) Gepinacin C) E1210.

Anchoring of proteins to the PM through covalent attachment to GPI provides an appealing potential target for the development of antifungal agents. Proteins designated to be GPI anchored have an *N*-terminal signal sequence for localization to the ER and a *C*-terminal signal sequence for attachment of the GPI anchor. An inhibitor of GPI-anchored wall protein transfer 1 (Gwt1), a critical acyltransferase required for the biosynthesis of fungal GPI anchors, is a potential antifungal drug target (Tsukahara et al., 2003). 1-[4-Butylbenzyl] isoquinoline (BIQ) was identified as an inhibitor of the surface expression of GPI-anchored mannoproteins in *Saccharomyces cerevisiae* and *C. albicans* (Fig. 5A). It was demonstrated that the Gwt1 protein, involved in inositol acylation of GPI biosynthesis, is a target of BIQ (Umemura et al., 2003). This compound also showed the ability to block adherence of *C. albicans* cells to mammalian epithelial cells and inhibits the cell wall assembly of GPI- anchored mannoproteins, leading to impaired cell growth in both *S. cerevisiae* and *C. albicans*.

Another small molecule, gepinacin (Fig. 5B) selectively inhibits Gwt1, without having effect on the viability of mammalian cells or inhibiting their orthologous acyltransferase (McLellan et al., 2012). Gepinacin was shown to increase the immunogenicity of *C. albicans*, further enhancing its antifungal activity by reducing the mannoprotein outer layer of the cell wall and unmasking the immunogenic inner β-glucan layer. This resulted in increased recognition of *Candida* by mammalian immune cells. Immunostaining and fluorescence microscopy revealed sublethal concentrations of gepinacin dramatically increased β-glucan presentation on the cell surface of *C. albicans*.

E1210, 3-(3-{4-[(pyridine-2-yloxy)methyl]benzyl}isoxazol-5-yl)pyridine-2-amine (Fig. 5C), acts as an inhibitor of the inositol acylation activity of *C. albicans* Gwt1p, which inhibits the synthesis of GPI and hampers the attachment of vital adhesion proteins, such as mannoproteins to the outer fungal cell wall. E1210 has shown good efficacy in the treatment of azole and echinocandin-resistant *C. albicans* strains (Miyazaki et al., 2011).

### β-(1,3)-D-Glucan synthase inhibitor

β-(1,3)-D-Glucan synthase (GS) is a vital enzyme in the synthesis of the essential component of the fungal cell wall, β-(1,3)-D-glucan. Cells exposed to echinocandin B (shows morphological changes such as swelling and release of intracellular contents (Eshwika et al., 2013). Echinocandins act as specific, non-competitive inhibitors of GS. As the cell wall is absent in humans/human cells, selective toxicity advantageous for GS inhibitors but the development of resistance to echinocandins, particularly in *Candida* spp., is concerning. The resistance mechanisms involve amino acid substitutions and naturally occurring polymorphisms in hot-spot regions of FKs subunits of glucan synthase, cellular stress responses and increased chitin synthesis which decrease the sensitivity of the enzyme to drug (Perlin, 2015).

**Fig. 6:**
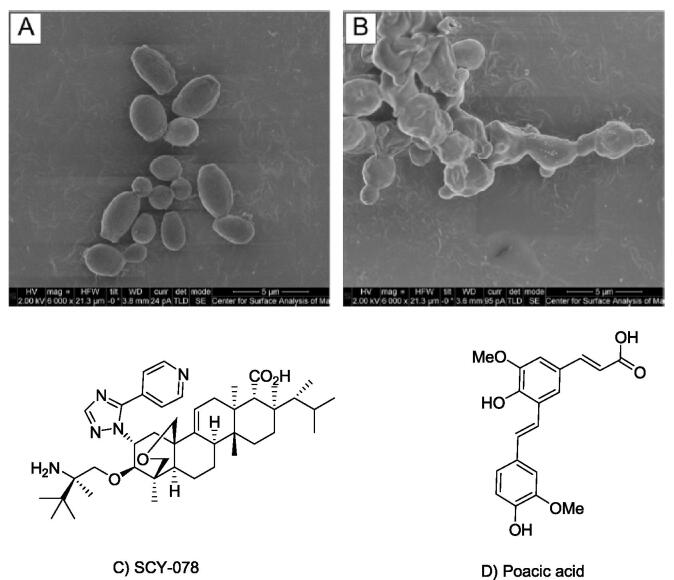
Scanning electron micrograph of *C. auris* treated with no drug (control) A) and with SCY-078 at 1 × MIC (0.5 mg/L) B), The untreated control *C. auris* cells showed a well-defined, oval shaped morphology as well as several budding cells. In contrast, cells exposed to SCY-078 showed a severely distorted cell topography with cells fused together, indicating they were unable to divide; Chemical structure of SCY-078C) [taken from (Larkin et al., 2017) using open access article under creative commons attribution (CC-BY 4.0) license].

To overcome the limitations of echinocandins, the effect of orally bioavailable triterpene, SCY-078 (Fig. 6C), a glucan synthase inhibitor (GSI) was assessed on growth, ultrastructure and biofilm forming ability of *C. auris* (Larkin et al., 2017). SCY-078 has been shown to exhibit both *in vivo* and *in vitro* activity against common *Candida* species and is currently the only β-(1,3)-glucan synthase inhibitor with both oral and intravenous treatment formulations in development (Berkow et al., 2017). SCY-078 had an MIC_90_ of 1.0 µg/mL against *C. auris* and causes complete inhibition of growth. Scanning electron microscopy showed that SCY-078 disrupted the ultrastructure of *C. auris* (Fig. 6A and B).

### *Candida* adhesion inhibition

**Fig. 7:**
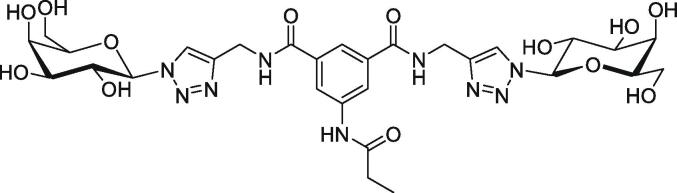
Chemical structure of anti-adherence divalent galactosyl AGC.

As mentioned earlier, adhesion is vital for establishing the *Candida*-host interaction. To infect its host, *Candida* cells adhere to the host cell using adhesions such as agglutinin-like sequence (ALS) proteins (Hoyer et al., 1995) and Hwp1 (Sundstrom et al., 2002). After initial adherence, contact with the host cell prompts the yeast-to-hypha transition, directed growth of the hypha takes place through thigmotropism. Invasins, adhesion and physical forces facilitate invasion into the host cell which occurs *via* endocytosis or active penetration (Gow et al., 2012). *Candida albicans* binds to glycans at the surface of epithelial cells to initiate infection. Aromatic glycoconjugates (AGCs) are small molecules that inhibit *C. albicans* adherence to buccal epithelial cells (BECs). The divalent galactoside with triazolyl group directly linked to the anomeric position (Fig. 7) (as a very effective inhibitor of fungal adhesion), built on aromatic molecular scaffolds, is capable of displacing over 50% of yeast cells already attached to the BECs (Martin et al., 2018). Fluorescence imaging indicates that divalent galactosides may bind to structural components of the fungal cell wall.

Lysine-containing cyclodecapeptides are known as regioselectively addressable functionalized templates (RAFT) and are used as stable scaffolds for the *de novo* design of peptidomimics. Cyclopeptide and dendrimeric polylysine scaffolds are also used to form multivalent displays of the divalent galactoside glycomimetic (Fig. 7) (Martin et al., 2021). Adhesion assay studies showed that tetravalent polylysine glycodendrimers achieved better results than divalent galactosides, inhibiting the fungal adhesion by 64% based on observed scanning electron microscopy (SEM) imaging of the morphology of *C. albicans* cell after 24 h exposure.

### Peptidyl nucleoside antifungals

A variety of natural products with antibiotic activities, including molecules with polyketide frameworks (erythromycins and tetracyclines), oligosaccharide frameworks (aminoglycosides) and those with peptide scaffolds, are made by microorganisms (Walsh and Zhang, 2011). Microorganisms can also join nucleosides to peptide scaffolds to generate peptidyl nucleosides, which function as antibiotics. Uridine-based nikkomycins and polyoxins are examples of peptidyl nucleosides and are fungal chitin synthase inhibitors with fungistatic activities (Cabib, 1991).

#### Nikkomycins

**Fig. 8:**
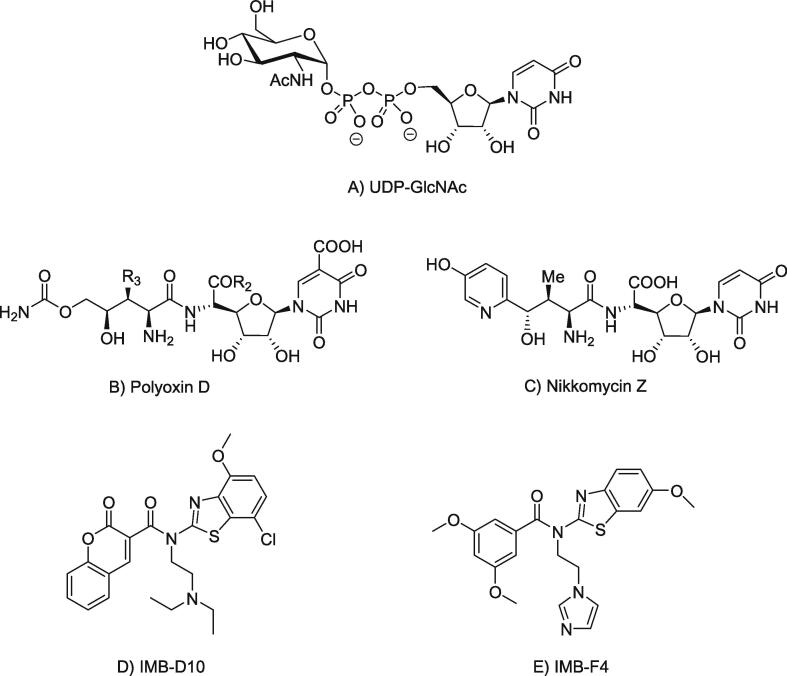
Chemical structure of A) UDP-GlcNAc, substrate of chitin biosynthesis B) Uridine-based Nikkomycin as fungal chitin synthase inhibitor C) IMB-D10 and D) IMB-F4 inhibit chitin synthase activity against *Candida albicans.*

Nikkomycins from *Streptomyces* strains contain two unusual amino acids, hydroxypyridylhomothreonine and 5-amino-5-deoxy-hexuronic acid, with an *N*-glycosidically linked base (Ginj et al., 2005). Nikkomycin Z (Fig. 8B), a chitin synthase inhibitor, has shown potential for therapeutic impact against *Candida* infections (Chapman et al., 1992). The therapeutic potential of nikkomycin Z combined with an echinocandin (anidulafungin or micafungin) was evaluated against two *Candida albicans* isolates (ATCC 90,028 and blood culture isolate CA 46503) and laboratory-derived echinocandin-resistant fks mutants (FKS1 S645Y and FKS1 S645P). Even though echinocandins are established as a front-line treatment for invasive *Candida* infections, monotherapy with nikkomycin Z prolonged the survival of infected mice, but the survival rates declined when nikkomycin Z treatment was stopped. The fks mutants, derived from the two *C. albicans* parent strains, were homozygous and harbored a single substitution mutation in the fks1 hot-spot region. According to the fractional inhibitory concentration index (FICI) and minimum inhibitory concentration (MIC) results, the fks mutants showed 32-fold elevations in MIC for anidulafungin and micafungin. Synergistic effects with nikkomycin Z and echinocandin were observed in the parent strains and the fks mutants. A combination treatment with nikkomycin Z and echinocandin showed significantly improved survival rate of mice infected with the *fks* mutants compared with that of mice treated with nikkomycin Z or echinocandin monotherapy. Only one dosing regimen for nikkomycin Z (at 10 mg/ml twice daily) was reported, which was the limitation of this study (Cheung and Hui, 2017).

### Chitin synthesis inhibition by a chemical-genetic method

Chitin is one of the essential components of the fungal cell wall. Three chitin synthases, CaChs1p, CaChs2p, and CaChs3p, in *C. albicans* are structurally and functionally analogous to those of *S. cerevisiae* (Sudoh et al., 2000). They are encoded by genes called *CaCHS1*, *CaCHS2* and *CaCHS3*. *CaCHS1* is essential for the viability of *C. albicans* and Cachs2p is the most abundant protein among the three chitin synthases, but it is not vital for viability, hyphal growth or virulence. Both CaChs1p and CaChs3p are required for septum formation and cell wall synthesis. The fourth chitin synthase in *Candida albicans*, *CHS8* produces class I enzyme like that of *CHS2* and differs from *S. cerevisiae* in having two isoenzymes in this class (Munro et al., 2003).

A chemical-genetic method is an approach used to isolate (screen) antifungal agents that target chitin synthesis in fungal cells by impairing chitin synthesis (Li et al., 2019). Two benzothiazoles (IMB-D10 and IMB-F4) (Fig. 8 C and D) have been reported to act as inhibitor of chitin synthesis and reducing chitin level in fungal cells (Lucero et al., 2002). These compounds showed higher level of toxicity to fungal mutants lacking glucan synthase FKs1 compared to wild type (WT) fungal cells and mutants lacking chitin synthase CaChs3p. Both inhibit the activity of chitin synthase in vitro. IMB-D10 inhibits CaChs1p activity, reduces the protein level of the three chitin synthases and shows higher inhibitory activity against *C. albicans* than IMB-F4.

## Anti-fungal vaccine development

Despite advances in medicine, there are no licensed vaccines available to prevent and control human fungal infections (Oliveira et al., 2021). Reasons for the lack of clinically available fungal vaccines include the very high costs related to manufacturing of the antigens, toxicology issues and the high cost of conducting clinical trials to establish tolerability and safety. In addition, challenges to developing a vaccine lie in translating preclinical studies in animals to humans.

### Live-attenuated fungal vaccines

Vaccines composed of whole organism (live-attenuated or killed fungal cells) show high immunogenicity, which makes them most likely to generate protective response but there is a potential risk of infection. Therefore, inactivated organism and subunit vaccines are safer but immunocompromised individuals are less likely to respond to vaccination. Edward Jenner’s vaccinia virus (the first ever reported vaccine) was a live-attenuated vaccine, which caused only a very mild form of the disease in humans while at the same time presenting long-lasting protective immune memory (Wan Tso et al., 2018).

### Recombinant proteins

In contrast to a prospect of live *Candida* vaccines, recombinant vaccines do not contain any infectious agent and are safer to the human host (Wan Tso et al., 2018). This characteristic makes recombinant vaccines suitable for immunocompromised individuals. This strategy focuses on proteins expressed on the surface of the fungus, such as cell wall proteins (CWPs) or adhesion proteins, to ensure that epitopes would be easily accessible to the immune system.

One of the recombinant *Candida* vaccines, PEV7, has reached human clinical testing with promising results (Oliveira et al., 2021). PEV7 consists of recombinant aspartyl-proteinase 2 (Sap2), a secreted protein of *Candida albicans*, assembled into virosomes. Following observed protection in *C. albicans*-challenged rat model study, a phase 1 clinical trial was conducted to assess the safety and immunogenicity of PEV7 in 48 healthy female volunteers. All of the vaccinated women established specific B-cell memory responses (ClinicalTrials.gov identifier: NCT01067131) (De Bernardis et al., 2018).

### Synthetic carbohydrate antigens

**Fig. 9:**
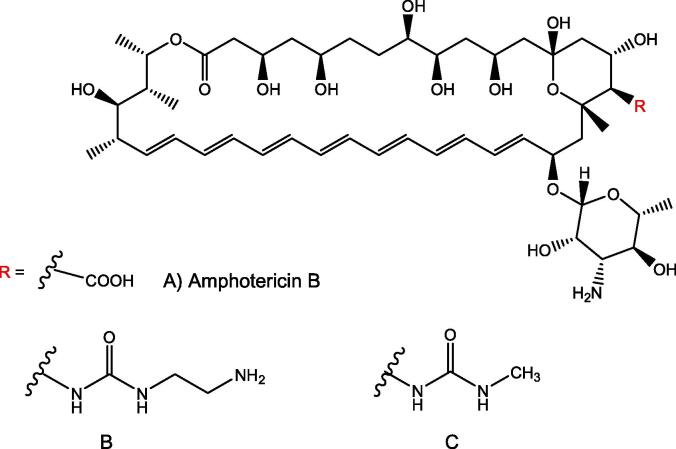
Structure of synthetic glycopeptide vaccine combining β-mannan and peptide epitope shows protection against candidiasis.

Unconjugated oligosaccharides are poorly immunogenic but conjugation to a protein carrier enhances their immunogenicity (Xin et al., 2008). Glycoconjugates from the capsular polysaccharide of pathogenic bacteria and carrier proteins, such as tetanus or diphtheria toxoids, have been used as effective vaccines (Bundle et al., 2018). These vaccines activate T-cells and are effective in reducing the incidence of dangerous infectious. Systematic induction of a secondary immune response and the recruitment of T helper cells are crucial factors in the success of conjugate vaccines. A fully synthetic glycopeptide vaccine (Fig. 9) has been constructed from a β-(1,2)-linked mannotriose conjugated to a T-cell peptide to obtain the glycopeptide conjugates, which demonstrate protection against *C. albicans* (Xin et al., 2008).

It was previously thought that the oligosaccharide component of conjugate vaccines required ≥ 10 residues, but recent studies prove that conjugate vaccines composed of shorter oligosaccharide epitopes are also protective. A vaccine candidate derived from a synthetic β-(1,2)-linked mannose oligosaccharide hapten conjugated to tetanus toxoid could induce high titer antibody, capable of opsonizing the surface of *C. albicans* cells after two injections in an immunocompromised rabbits model (Lipinski et al., 2012). Significant reduction of fungal burden in animals’ vital organs exhibited after vaccination. These results support that this synthetic vaccine was a promising approach to combat fungal *Candida* infections.

## Non-medical applications

### Plant fungal pathogens and inhibitors

In plant-fungus interaction, conserved and specific cell wall polysaccharides of various fungi, such as chitin are recognised as PAMPs *via* pattern recognition receptor (PRRs) in plants. When fungal pathogen invade host plants, recognition of PAMPs triggers immunity in plants (PTI). Various cellular defense responses in plants are associated with PTI, including generation of reactive oxygen species, reinforcement of the plant cell wall and changes in gene expression (Nishimura, 2016).

**Fig. 10:**
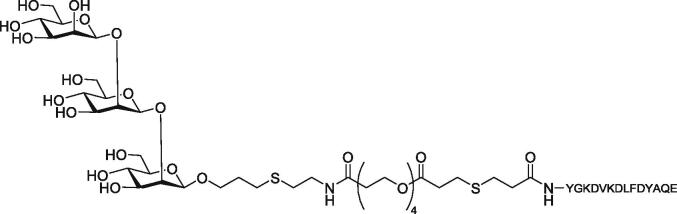
Chemical structure of A) *Neurospora crassa* inhibitor, Aminopyrifen B) plant natural product poacic acid, C) Uridine-based fungal chitin synthase inhibitor, polyoxin D.

Plant diseases cause serious loss of crop production and it is crucial to develop novel fungicides to protect crops from fungal plant pathogens. Aminopyrifen (Fig. 10A), 4-phenoxybenzyl 2-amino-6-methylnicotinate, showed antifungal activity against *Neurospora crassa* and mycelial growth inhibition was detected at very low concentrations on Vogel’s medium containing 1.2% sucrose, with a 0.001 mg/L concentration required for 50% growth inhibition (Hatamoto et al., 2019). To identify the target protein for aminopyrifen, resistant mutants were isolated and the resistance mutations were localized to the *gwt-1* gene that encodes Gwt1 and were found to result in S180F and V178A alterations in the protein. These results suggest that aminopyrifen acts as an inhibitor of Gwt1, a protein involved in GPI-anchored biosynthesis.

A plant natural product, poacic acid (PA) (Fig. 10B), found in lignocellulosic hydrolysates of grasses, has shown to have antifungal activity against a range of plant pathogens and against *Saccharomyces cerevisiae*. PA has been reported to target β-(1,3)-glucan synthesis within the fungal cell wall (Piotrowski et al., 2015). Poacic acid causes quick cell lysis and is synergistic with antifungal drugs that target the cell wall and membrane integrity, such as fluconazole and caspofungin. PA also shows a species-dependent effect on the human fungal pathogens from the genus *Candida*. Unlike the echinocandins, it inhibits β-(1,3)-glucan by directly binding to β-(1,3)-glucan polysaccharide rather than inhibiting glucan synthase. (Lee et al., 2018). PA sensitivity is controlled by the Ca^2+^ / calcineurin pathway and susceptibility to PA varies between *Candida* species. The inhibitor effect of PA varies in a range of *Candida* pathogens inhibition, which limits its potential for development as new antifungal agent.

The polyoxins are produced by *Streptomyces* spp. and exhibit powerful bioactivity against phytopathogenic fungi. Polyoxin is known to act as a competitive inhibitor of the chitin synthetase (Jackson et al., 2013) as its structure mimics that of UDP-*N*-acetylglucosamine (UDP-GlcNAc), a substrate for chitin biosynthesis. One of the major components of the polyoxin group, polyoxin D (Fig. 10C), is active against plant invaders such as the fungus *Cochliobolus miyabeanus* that causes severe leaf spot disease on rice and two North American specialty crops, American wildrice and switchgrass (Castell-Miller et al., 2016). Studies showed that polyoxin D selectively inhibits the incorporation of ^14^C-glucosamine into the cell wall chitin of *Neurospora crassa* by approximately 50%; levels comparable to those required for fungal cell death (Endo et al., 1970). This nucleoside derivative also increases accumulation of UDP-GlcNAc, further demonstrating inhibition of chitin synthesis.

## Conclusions

In conclusion, this review summarizes the fungal cell wall composition across a range of *Candida* spp. with the focus on synthesis of β-glucan and chitin, the core polysaccharides where synthesis is initiated at the plasma membrane. Furthermore, the biosynthesis of fungal cell wall proteins are described, as they play a role in modifying the cell wall properties for adherence to surfaces and protecting the fungal from microenvironment. For decades, a variety of fungal cell wall antigens (and non-cell wall antigens) have been evaluated in preclinical studies and most of these works have been done in mouse models of vaccination, followed by challenge of the fungal organism. Three major classes of antifungal agents have already been established. However, there is scope for direct antifungal activity of novel agents such as small molecules, live-attenuated, recombinant and synthetic vaccines, which could help address the challenge of drug-resistant fungi in the near future.

## Declaration of Competing Interest

The authors declare that they have no known competing financial interests or personal relationships that could have appeared to influence the work reported in this review.
